# Magnetoelastic thin films at large strains

**DOI:** 10.1007/s00161-020-00904-1

**Published:** 2020-08-01

**Authors:** Elisa Davoli, Martin Kružík, Paolo Piovano, Ulisse Stefanelli

**Affiliations:** 1 Institute of Analysis and Scientific Computing, TU Wien, Wiedner Hauptstrasse 8-10, 1040 Vienna, Austria; 2 Czech Academy of Sciences, Institute of Information Theory and Automation, Pod Vodárenskou věží4, 180 00 Prague, Czechia; 3 Faculty of Mathematics, University of Vienna, 1090 Vienna, Austria; 4 Vienna Research Platform on Accelerating Photoreaction Discovery, University of Vienna, Währingerstraße 17, 1090 Vienna, Austria; 5 Istituto di Matematica Applicata e Tecnologie Informatiche E. Magenes, Via Ferrata 1, 27100 Pavia, Italy

**Keywords:** Magnetoelasticity, Thin-films, Eulerian–Lagrangian, Formulations, Large-strain deformations

## Abstract

Starting from the three-dimensional setting, we derive a limit model of a thin magnetoelastic film by means of $\varGamma$-convergence techniques. As magnetization vectors are defined on the elastically deformed configuration, our model features both Lagrangian and Eulerian terms. This calls for qualifying admissible three-dimensional deformations of planar domains in terms of injectivity. In addition, a careful treatment of the Maxwell system in the deformed film is required.

## Introduction

Magnetoelasticity describes the mechanical behavior of solids under magnetic effects. The magnetoelastic coupling is based on the presence of small magnetic domains in the material [13]. In the absence of an external magnetic field, these magnetic domains are randomly oriented. When an external magnetic field is applied, the mesostructure of magnetic domains changes by magnetic-domain wall motion, by magnetization-vector rotation, and, for some specific alloys, by magnetic-field-driven martensitic-variant transformation. The net effect is a magnetically induced deformation in the body. Conversely, mechanical deformations modify the magnetic response of a specimen by influencing the magnetic anisotropy of the domains, so that the magnetic and the mechanical behavior of the material are fully coupled. We refer to, e.g., [4, 7, 8, 14] for an exposition on the foundations of magnetoelasticity and to [17] for some related mathematical considerations.

The mathematical modeling of magnetoelasticity is a lively area of research, triggered by the interest in the so-called *multifunctional* materials. Among these, one has to mention rare-earth alloys such as TerFeNOL and GalFeNOL, as well as ferromagnetic shape-memory alloys as $\hbox {Ni}_2$MnGa, NiMnInCo, NiFeGaCo, FePt, FePd, among others [16]. These materials exhibit a remarkable magnetostrictive behavior, for reversible strains as large as 10% can be activated by the imposition of relatively moderate magnetic fields. This strong magnetoelastic coupling makes them relevant in a wealth of innovative applications including sensors and actuators [2].

The aim of this paper is to present a model of a thin film undergoing large strain deformations in the membrane regime. This will be inferred from a variational dimension-reduction procedure from a corresponding three-dimensional model at large strains.

Dimension-reduction techniques play an important role in nonlinear analysis and numeric s, for they allow simpler computational approaches, still preserving the main features of the corresponding bulk model. The last decades have witnessed remarkable progresses on dimension reduction by variational methods, particularly by $\varGamma$-convergence [3, 6], together with quantitative rigidity estimates [9]. Among the many results on the elastic response of low-dimensional objects, we mention the rigorous justification of membrane theory [22, 23], bending theory [9, 27], and von Kármán theory [10, 21] for plates as variational limits of nonlinear three-dimensional elasticity for vanishing thickness. In particular, we refer to [10] for the derivation of a hierarchy of different plate models and for a thorough literature review.

A rigorous derivation of a model for magnetic thin films has been first obtained in [11]. A rate-independent evolution of Kirchhoff–Love magnetic plates together with the passage from three-dimensional linearized magnetoelasticity to the corresponding two-dimensional theory is the subject of [19]. Magnetostriction in thin films has been considered, also from the numerical viewpoint, in [24–26]. With respect to these results, this paper presents a fundamental novelty as it represents the first rigorous analytical treatment including also the *large-strain* magnetoelastic regime.

In the classical dimension reduction for *small-strain* elastic thin plates, the analysis is set in cylindrical domains whose heights depend on a thickness parameter eventually tending to zero. The same setting applies in magnetoelasticity. Under the small-deformations assumption, the magnetization may be assumed to be directly defined on the reference configuration. This simplification is, however, not amenable in the large-strain regime, for the magnetization is defined on the *deformed* configuration instead. The latter is, however, a priori not known, as it depends on the deformation itself. In particular, this naturally leads to a mixed Eulerian–Lagrangian formulation of the problem. Compared with previous small-strain contributions, the mathematical framework of this work is hence much more involved. A distinctive difficulty arises from the need of ensuring that admissible deformations are globally injective. In the bulk, this can be achieved by imposing the so-called Ciarlet–Nečas condition [5]. For films, however, no comparable condition, i.e., allowing for a variational approach, seems to be available. A further difficulty is represented by the Maxwell system, which is formulated in actual space. In order to identify the asymptotic behavior of the stray field, we have to characterize the limiting differential constraints in weak form by keeping track of the deformed configuration.

The main result of the paper is the derivation of a variational model for thin-film specimens as a $\varGamma$-limit of a suitably scaled energies of a bulk model for vanishing thickness. In Theorem 3.2, we prove in full generality the $\varGamma$–$\liminf$ inequality, showing that our limit energy functional always represents a lower bound for the asymptotic behavior of the three-dimensional energy functionals. If the limit film deformation is *approximately injective* in the sense of Definition 3.3, we show that the $\varGamma$–$\liminf$ is indeed the largest lower semicontinuous lower bound for the magnetoelastic-plate functionals as the thickness goes to zero, i.e., it is the $\varGamma$-limit; cf.  Theorem 3.4. Here, the approximate injectivity means that there is a sequence of deformations of the bulk which are globally injective and converge in a suitable sense to the film deformation. Additionally, in Theorem 3.5 we prove a complete $\varGamma$-convergence result under the additional assumption that the admissible three-dimensional deformations satisfy a suitable injectivity requirement which guarantees that the limit deformation of the film is globally injective.

The paper is organized as follows. In Sect. 2, we introduce the mathematical setting of the problem. Section 3 is devoted to the statements of all results, and Sect. 4 contains all proofs.

## Setting of the problem

We use the standard notation for Sobolev and Lebesgue spaces, i.e., $$W^{k,p} $$ and $$L^p $$ [1]. If $$A\in {\mathbb {R}}^{3\times 2}$$ and $$b\in {\mathbb {R}}^3$$ we write $$(A|b)\in {\mathbb {R}}^{3\times 3}$$ for a matrix whose first two columns are created by the first two columns of *A* and the third one by the vector *b*. The set of proper rotations is denoted by SO$$(3):=\{R\in {\mathbb {R}}^{3\times 3}:\, R^\top R= RR^\top = \text {Id}, \ \mathrm{det}\, R=1\}$$ where Id is the identity matrix.

Let $\omega \subset {\mathbb {R}}^2$ be a bounded Lipschitz domain representing the planar reference configuration of the film, define the reference configuration of a thin magnetoelastic plate as

$$\begin{aligned} \varOmega _h:=\omega \times \Big (-\frac{h}{2},\frac{h}{2}\Big ), \end{aligned}$$

and set $\varOmega :=\varOmega _1$. In the expression above, $h>0$ represents the thickness of the plate, eventually bound to go to zero. Correspondingly, we will consider limits as $h \rightarrow 0$ of sequences of functionals by means of $\varGamma$-convergence [6]. This is a standard approach to characterize the limiting behavior of a sequence of bulk energies for specimens of very small thickness.

Assume that *X* is a subset of a reflexive Banach space. We say that $\{I_h\}_{h>0}$ for $I_h:X\rightarrow {\mathbb {R}}\cup \{\infty \}$ $\varGamma$-converges to $I:X\rightarrow {\mathbb {R}}\cup \{+\infty \}$ if the following conditions hold simultaneously:

2.1a $$\begin{aligned}&\zeta _h{\mathop {\rightarrow }\limits ^{X}} \zeta \ \Rightarrow \ \liminf _{h\rightarrow 0}I_h(\zeta _h)\ge I(\zeta ), \end{aligned}$$

2.1b $$\begin{aligned}&\forall \, \zeta \in X\ \exists \{{\hat{\zeta }}_h\}_{h>0}\subset X: \quad {\hat{\zeta }}_h{\mathop {\rightarrow }\limits ^{X}} \zeta \ \text {and} \ \limsup _{h\rightarrow 0}I_h({\hat{\zeta }}_h)=I(\zeta ), \end{aligned}$$

where the symbol ${\mathop {\rightarrow }\limits ^{X}}$ indicates the convergence with respect to a properly chosen (weak) topology in *X*. If (2.1) holds, we say that *I* is the $\varGamma$-limit of $\{I_h\}_{h>0}$ (with respect to that topology).

The state of the magnetoelastic material is defined in terms of its *deformation* *w* and its magnetization *m*. The deformation $w: \varOmega _h \rightarrow {\mathbb {R}}^3$ is required to belong to $W^{1,p}(\varOmega _h;{\mathbb {R}}^3)$ for some given

$$\begin{aligned} p>3, \end{aligned}$$

to be orientation-preserving, namely, $\det \nabla w >0$ almost everywhere, and to satisfy the Ciarlet–Nečas condition [5]

2.2 $$\begin{aligned} \int _{\varOmega _h} \mathrm{det }\,\nabla w \,\mathrm{d}x\le {\mathcal {L}}^3(w(\varOmega _h)) \end{aligned}$$

where ${\mathcal {L}}^3$ stands for the three-dimensional Lebesgue measure. In particular, *w* is identified with the unique continuous representative in the equivalence class. The magnetization *m* is set on the open deformed configuration, namely, $m:\varOmega ^w_h \rightarrow {{\mathbb {S}}}^2$, where $\varOmega ^w_h$ is given by

$$\begin{aligned} \varOmega _h^w:=w(\bar{\varOmega }_h){\setminus } w(\partial \bar{\varOmega }_h) \end{aligned}$$

which is well-defined, for *w* is continuous. The magnetization *m* is hence required to fulfill the *saturation* constraint $|m|=1$ on $\varOmega _h^w$.

In what follows, for every $x\in {\mathbb {R}}^3$ in the referential space we write $x=(x',x_3)$ where $x'\in {\mathbb {R}}^2$ is referred to as the planar coordinates of *x*, and we denote by $\nabla '$ the gradient with respect to such planar coordinates. We use instead the symbol $\xi \in {\mathbb {R}}^3$ to indicate variable s in the actual space.

Following the approach in [15, 20, 28], we consider the total energy $I_h$ defined as

2.3 $$\begin{aligned} I_h(w,m)&:=\int _{\varOmega _h}W(\nabla w(x),m\circ w(x))\mathrm{d}x+\alpha \int _{\varOmega _h^{w}}|\nabla m(\xi )|^2 \mathrm{d}\xi +\int _{\varOmega _h}|\nabla ^2w(x)|^p\,\mathrm{d}x\nonumber \\&\quad +\int _{\varOmega _h}\varPhi (\nabla w(x))\mathrm{d}x+\frac{\mu _0}{2}\int _{{\mathbb {R}}^3}|\nabla u_m(\xi )|^2\,\mathrm{d}\xi . \end{aligned}$$

In the formula above, $W:\mathbb {M}^{3\times 3}\times \mathbb {S}^2\rightarrow [0,+\infty )$ is the *elastic energy* density associated with the plate, which is a continuous function satisfying the following assumptions:

2.4 $$\begin{aligned}&\text {(Coercivity)}\quad \exists c>0 \text { such that }W(F,\lambda )\ge c|F|^p-\frac{1}{c}, \end{aligned}$$

2.5 $$\begin{aligned}&\text {(Frame indifference)}\quad \ W(RF,R\lambda )=W(F,\lambda ), \end{aligned}$$

2.6 $$\begin{aligned}&\text {(Magnetic parity)}\quad \quad \,W(F,\lambda )=W(F,-\lambda ) \end{aligned}$$

for every $F\in \mathbb {M}^{3\times 3}$, $R\in \mathrm{SO}(3)$, and $\lambda \in \mathbb {S}^2$. In fact, assumptions (2.5)–(2.6) are not strictly needed for the analysis, but rather required by modeling considerations.

The second term in the expression of $I_h$ in (2.3) is the *exchange energy*. The constant $\alpha$ is related to the size of ferromagnetic texture. The material is assumed to be of *nonsimple* type [18]. This is expressed by the occurrence of the third term in $I_h$, providing a higher-order contribution and a further length scale to the problem. Regarding the fourth term, we will require that $\varPhi :\mathbb {M}^{3\times 3}\rightarrow [0,+\infty )$ is a continuous map satisfying the following assumptions

2.7 $$\begin{aligned}&\varPhi (F)\rightarrow +\infty \quad \text {as }\,\mathrm{det}\,F\rightarrow 0^+,\nonumber \\&\varPhi (F)=+\infty \quad \text {if }\,\mathrm{det}\,F\le 0,\nonumber \\&\varPhi (F)\ge \frac{1}{C}(\mathrm{det}\,F)^{-q}\quad \text { for some } C>0\text { and for every }\,F\in \mathbb {M}^{3\times 3}\,\text {with }\mathrm{det}\,F>0, \end{aligned}$$

where $q>\frac{3p}{p-3}$. This last quantification is introduced in [12] and ensures that, for all $\lambda >0$ and $w\in W^{2,p}(\varOmega _h;{\mathbb {R}}^3)$ such that

2.8 $$\begin{aligned} \int _{\varOmega _h}|\nabla ^2 w(x)|^p\,\mathrm{d}x+\int _{\varOmega _h}\varPhi (\nabla w(x))\mathrm{d}x < \lambda \end{aligned}$$

there exists $c>0$ depending on $\lambda >0$ with the property that

$$\begin{aligned} \mathrm{det }\,\nabla w>c\quad \text { in }{\bar{\varOmega }}_h. \end{aligned}$$

Note that the left-hand side of inequality (2.8) is a part of the energy functional (2.3). The last term in (2.3) represents the *magnetostatic energy*. In particular, $\mu _0$ is the *permittivity* of void, and $u_m$ solves the Maxwell equation

$$\begin{aligned} \nabla \cdot (-\mu _0\nabla u_m+\chi _{\varOmega _h^w}m)=0\quad \text {in }{\mathbb {R}}^3, \end{aligned}$$

where $\chi _{\varOmega _h^w}$ is the characteristic function of the set $\varOmega _h^w$. For simplicity, we assume that the deformations *w* satisfy the boundary conditions

$$\begin{aligned} w =\text {id}\quad \text {and}\quad \nabla w=\text {Id}\quad \text {on }\,\partial \omega \times \big (-\tfrac{1}{2},\tfrac{1}{2}\big ). \end{aligned}$$

To consider alternative boundary conditions would call for solving some additional technicalities which, we believe, would excessively complicate the argument. We hence leave this extension to some possible further investigation.

### Change of variables

As customary in dimension reduction, we perform the change of variables

$$\begin{aligned} \phi _h:\varOmega \rightarrow \varOmega _h,\quad \phi _h(x):=(x_1,x_2,hx_3)\quad \text {for a.e. }x\in \varOmega . \end{aligned}$$

Setting $y:=w\circ \phi _h$, $\varOmega ^y := y (\bar{\varOmega }){\setminus } y(\partial {\bar{\varOmega }})$, and $E^h(y,m):=\frac{1}{h}I^h(w,m)$, we obtain

$$\begin{aligned} E_h(y,m)&:=\int _{\varOmega }W(\nabla _hy(x),m\circ y(x))\mathrm{d}x+\frac{\alpha }{h}\int _{\varOmega ^y}|\nabla m(\xi )|^2\mathrm{d}\xi +\int _{\varOmega }|\nabla ^2_h y(x)|^p\,\mathrm{d}x\nonumber \\&\quad +\int _{\varOmega }\varPhi (\nabla _h y(x))\,\mathrm{d}x+ \frac{\mu _0}{2h}\int _{{\mathbb {R}}^3}|\nabla u_m(\xi )|^2\mathrm{d}\xi , \end{aligned}$$

where

$$\begin{aligned} \nabla \cdot (-\mu _0\nabla u_m+\chi _{\varOmega ^y}m)=0\quad \text {in}{\mathbb {R}}^3. \end{aligned}$$

Above, $\nabla _h$ and $\nabla _h^2$ are the differential operators defined as

$$\begin{aligned}&\nabla _hv :=\Big (\partial _1v \big |\partial _2 v \big |\frac{\partial _3 v }{h}\Big ),\quad \text {and}\quad \nabla _h^2 v:=\Bigg (\begin{array}{ccc}\quad \partial ^2_{11}v&{}\quad \partial ^2_{21}v&{}h^{-1}\partial ^2_{31}v\\ \quad \partial ^2_{12}v&{}\quad \partial ^2_{22}v&{}h^{-1}\partial ^2_{32}v\\ h^{-1}\partial ^2_{13}v&{}h^{-1}\partial ^2_{23}v&{}h^{-2}\partial ^2_{33}v\end{array}\Bigg ) \end{aligned}$$

for every $v\in W^{2,p}(\varOmega )$. Note that the three-dimensional Ciarlet–Nečas condition becomes

2.9 $$\begin{aligned} \int _{\varOmega }\mathrm{det}\,\nabla _h y\,\mathrm{d}x\le \frac{{\mathcal {L}}^3(\varOmega ^{y})}{h}. \end{aligned}$$

Condition (2.9) provides scant information in the thin-film regime, for it leads to the inequality

$$\begin{aligned} \int _\omega (\partial _1 y\times \partial _2 y)\cdot b\, \mathrm{d} S\le \lim _{h\rightarrow 0} \frac{{\mathcal {L}}^3(\varOmega ^{y})}{h} \end{aligned}$$

where *b* is a *Cosserat* vector obtained as $b=\lim _{h\rightarrow 0} h^{-1} \partial _3 y^h$ in $W^{1,p}(\omega ;{\mathbb {R}}^3)$. In particular, if $b= (\partial _1 y\times \partial _2 y)/|\partial _1 y\times \partial _2 y|$, i.e., it is the unit normal vector to the film in the deformed configuration, and if $\lim _{h\rightarrow 0} \frac{{\mathcal {L}}^3(\varOmega ^{y})}{h}={\mathcal {H}}^2(y(\omega ))$ we get

2.10 $$\begin{aligned} \int _\omega |\partial _1 y\times \partial _2 y|\,\mathrm{d}S\le {\mathcal {H}}^2(y(\omega )). \end{aligned}$$

**Fig. 1:**
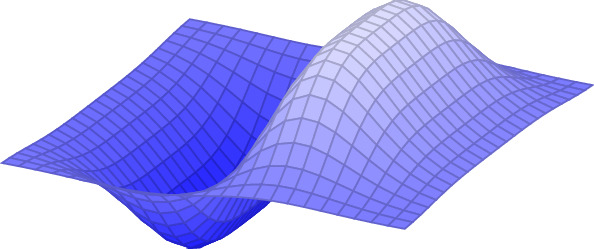
An injective deformation satisfying (2.10)

**Fig. 2:**
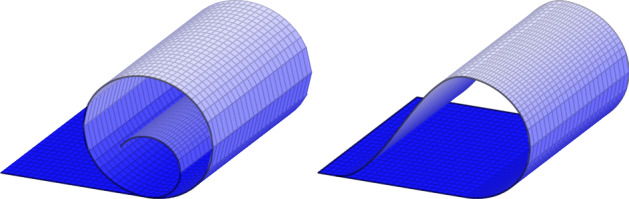
Two deformations not satisfying (2.10) in the regions in which a self-contact occurs

**Fig. 3:**
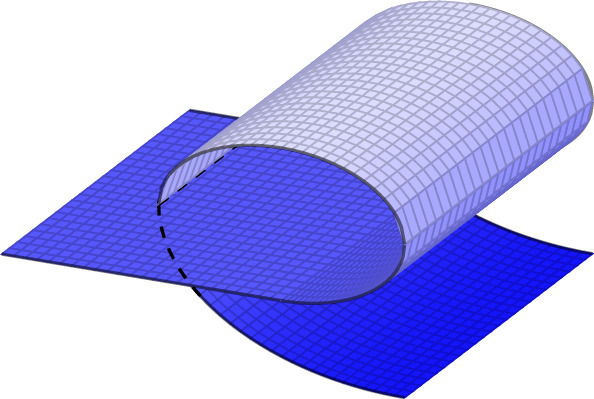
A self-interpenetrating deformation satisfying (2.10)

The left-hand side of (2.10) is the area of the deformed film calculated by the change-of-variables formula, while the right-hand side is the measured area. Hence, (2.10) is violated by a *folding* deformation, which should be admissible among the family of realistic thin-film deformations, while (2.10) is satisfied if the film crosses itself, which violates non-self-interpenetration of matter and is hence not admissible. On the other hand, if $y:\varOmega \rightarrow {\mathbb {R}}^3$ is injective then (2.10) is satisfied. The situation is depicted in Figs. 1, 2 and 3.

In what follows, we analyze the asymptotic behavior of sequences $(y^h,m^h)\in W^{2,p}(\varOmega ;{\mathbb {R}}^3)\times W^{1,2}(\varOmega ^{y^h};{\mathbb {R}}^3)$ satisfying the uniform energy estimate

2.11 $$\begin{aligned} E_h(y^h,m^h)\le C, \end{aligned}$$

and the boundary conditions

2.12 $$\begin{aligned} y(x)=(x',hx_3)\quad \text {and}\quad \nabla y(x)=\Bigg (\begin{array}{ccc}1&{}0&{}0\\ 0&{}1&{}0\\ 0&{}0&{}h\end{array}\Bigg )\quad \text {on }\,\partial \omega \times \big (-\tfrac{h}{2},\tfrac{h}{2}\big ). \end{aligned}$$

A *caveat* on notation: in (2.11) and in the following the symbol *C* is used to denote a generic constant that may possibly change from line to line and that always depends only on model data and not on *h*.

We point out that, without the $\varPhi$ term in the energy and the Ciarlet–Nečas condition, constant deformations *y* having null gradient, null hessian, and such that the measure of the deformed set is zero (so that the exchange energy gives no contribution) would be energetically favorable both for the elastic and the exchange energy. The associated magnetic field would then concentrate in a point. The $\varPhi$ term in our model prevents this degenerate situation from happening.

## Main results

This section is devoted to the statement of our main $\varGamma$-convergence results. All proofs are postponed to the following Sect. 4.

For notational convenience, for every open set $U\subset {\mathbb {R}}^2$ we denote by $\mathring{W}^{k,p}(U;{\mathbb {R}}^n)$ the set of $W^{k,p}$-maps having zero mean on *U*, i.e., $y\in \mathring{W}^{k,p}(U;{\mathbb {R}}^n)$ if $y\in W^{k,p}(U;{\mathbb {R}}^n)$ and $\int _U y(x')\,\mathrm{d}x'=0$. As it is standard, we write $\mathring{W}^{k,p}(U)$ if $n=1$.

We first introduce the set ${\mathcal {A}}$ of *admissible limiting deformations* $y:\omega \rightarrow {\mathbb {R}}^3$,* Cosserat vectors* $b:\omega \rightarrow {\mathbb {R}}^3$, *and magnetizations* ${\mathscr {M}}: \omega \rightarrow {\mathbb {S}}^2$, defined as

$$\begin{aligned}&{\mathcal {A}}:=\{(y,b,{\mathscr {M}}):\,y\in \mathring{W}^{2,p}(\omega ;{\mathbb {R}}^3),\,b\in W^{1,p}(\omega ;{\mathbb {R}}^3),{\mathscr {M}}\in W^{1,2}(\omega ;\mathbb {S}^2),\\&y=\text {id}\text { and }\big (\nabla ' y|b\big )=\text {Id}\text { on }\,\partial \omega ,\\&(\nabla 'y|b)^{-1}\in C^0(\bar{\omega };\mathbb {M}^{3\times 3}),\ \text {det}(\nabla 'y|b)\in C^0(\bar{\varOmega }), \ \text {and}\ \text {det}(\nabla 'y|b)>\varepsilon \ \text {for some }\varepsilon >0\}. \end{aligned}$$

Let us first state the following lemma, which will be instrumental in characterizing the limiting stray fields and formulating the limiting functional. As mentioned, the lemma is proved in Sect. 4 below.

### Lemma 3.1

Let $(y,b,{\mathscr {M}})\in {\mathcal {A}}$. Denote by $\widetilde{(\nabla 'y|b)}$ and $\bar{{\mathscr {M}}}$ the quantities

3.1 $$\begin{aligned} \widetilde{(\nabla 'y|b)}(x'):= {\left\{ \begin{array}{ll} (\nabla 'y|b)(x')&{}\text {if}\ x'\in \omega \\ \mathrm{Id}&{} \text {if}\ x'\in \mathbb {R}^2{\setminus }\omega . \end{array}\right. } \end{aligned}$$

and

3.2 $$\begin{aligned} \bar{{\mathscr {M}}}(x'):= {\left\{ \begin{array}{ll} {\mathscr {M}}(x')&{}\text {if}\ x'\in \omega \\ 0&{} \text {if}\ x'\in \mathbb {R}^2{\setminus }\omega . \end{array}\right. } \end{aligned}$$

Then, the system

3.3 $$\begin{aligned}&\Big \{\mathrm{cof}\,\widetilde{(\nabla ' y|b)^\top }\Big [\mu _0 \widetilde{(\nabla 'y|b)}^{-T}\Big (\nabla '{\mathscr {U}}|{\mathscr {V}}\Big )^T-\bar{{\mathscr {M}}}\Big ]\Big \}_3=0\quad \text {in }\,{\mathbb {R}}^2, \end{aligned}$$

3.4 $$\begin{aligned}&\mathrm{div}_{x'}\,\left\{ \begin{array}{c}\Big [\mathrm{cof}\,\widetilde{(\nabla ' y|b)^\top }\Big (\mu _0 \widetilde{(\nabla 'y|b)}^{-T}\Big (\nabla '{\mathscr {U}}|{\mathscr {V}}\Big )^T-\bar{{\mathscr {M}}}\Big )\Big ]_1\\ \Big [\mathrm{cof}\,\widetilde{(\nabla ' y|b)^\top }\Big (\mu _0 \widetilde{(\nabla 'y|b)}^{-T}\Big (\nabla '{\mathscr {U}}|{\mathscr {V}}\Big )^T-\bar{{\mathscr {M}}}\Big )\Big ]_2 \end{array}\right\} =0\quad \text {in }\,{\mathbb {R}}^2, \end{aligned}$$

has a unique solution $({\mathscr {U}},{\mathscr {V}})\in W^{1,2}({\mathbb {R}}^2)\times L^2({\mathbb {R}}^2)$ satisfying $\int _{\omega }{\mathscr {U}}\,\mathrm{d}x'=0$.

The limiting energy is given by the functional ${\mathcal {F}} :{\mathcal {A}}\rightarrow [0,+\infty )$ defined as

$$\begin{aligned} {\mathcal {F}}(y,b,{\mathcal {M}})&:=\int _{\omega }W\big ((\nabla ' y|b),\,{\mathscr {M}}\big )\,\mathrm{d}x'+ \alpha \int _{\varOmega }|(\nabla 'y|b)^{-T}(\nabla '{\mathscr {M}}|0)|^2\mathrm{det}\,(\nabla 'y|b)\,\mathrm{d}x\\&\quad +\int _{\omega }(|(\nabla ')^2 y|^2+2|\nabla ' b|^2)^{p/2}\,\mathrm{d}x'+\int _{\omega }\varPhi (\nabla 'y|b)\,\mathrm{d}x'\\&\quad +\frac{\mu _0}{2}\int _{\omega }\mathrm{cof}\,(\nabla 'y|b){\mathscr {M}}\cdot \Big (\nabla '{\mathscr {U}}_{y,b,{\mathscr {M}}}|{\mathscr {V}}_{y,b,{\mathscr {M}}}\Big )^T\,\mathrm{d}x' \end{aligned}$$

for every $(y,b,{\mathscr {M}})\in {\mathcal {A}}$, where the pair $\Big ({\mathscr {U}}_{y,b,{\mathscr {M}}}|{\mathscr {V}}_{y,b,{\mathscr {M}}}\Big )\in \mathring{W}^{1,2}(\omega )\times L^2(\omega )$ is the restriction to $\omega$ of the unique solution to (3.3)–(3.4) in the sense of Lemma 3.1.

We start by providing a lower bound for the asymptotic behavior of the functionals $\{E_h\}_h$ along sequences of deformations and magnetizations with equibounded energies. Again, the proof is postponed to Sect. 4.

### Theorem 3.2

(Compactness and $\varGamma$–$\liminf$ inequality) Let $\{(y^h,m^h)\}\subset W^{2,p}(\varOmega ;{\mathbb {R}}^3)\times W^{1,2}(\varOmega ^{y^h};{\mathbb {R}}^3)$ be such that (2.11) holds true. Then, there exist $(y,b,{\mathscr {M}})\in {\mathcal {A}}$ and $d\in L^p(\varOmega ;{\mathbb {R}}^3 )$ such that up to the extraction of a (not relabeled) subsequence there holds

3.5 $$\begin{aligned}&y^h\rightharpoonup y\quad \text {weakly in }W^{2,p}(\varOmega ;{\mathbb {R}}^3), \end{aligned}$$

3.6 $$\begin{aligned}&\nabla _h y^h\rightharpoonup (\nabla ' y|b)\quad \text {weakly in }W^{1,p}(\varOmega ;\mathbb {M}^{3\times 3}), \end{aligned}$$

3.7 $$\begin{aligned}&\frac{\partial ^2_{33}y^h}{h^2}\rightharpoonup d\quad \text {weakly in }L^p(\varOmega ;{\mathbb {R}}^3). \end{aligned}$$

Additionally, there exist $\eta \in L^2(\varOmega ;{\mathbb {R}}^3)$ and ${\mathscr {V}}\in L^2(\varOmega )$ such that $\int _{-\tfrac{1}{2}}^{\tfrac{1}{2}}{\mathscr {V}}\,\mathrm{d}x_3={\mathscr {V}}_{y,b,{\mathscr {M}}}$, and up to subsequences we have

3.8 $$\begin{aligned}&m^h\circ y^h\rightharpoonup {\mathscr {M}}\quad \text {weakly in }W^{1,2}(\varOmega ;{\mathbb {R}}^3), \end{aligned}$$

3.9 $$\begin{aligned}&\nabla _h(m^h\circ y^h)\rightharpoonup (\nabla '{\mathscr {M}}|\eta )\quad \text {weakly in }L^{2}(\varOmega ;\mathbb {M}^{3\times 3}), \end{aligned}$$

3.10 $$\begin{aligned}&u_{m^h}\circ y^h-\fint _{\varOmega }u_{m^h}\circ y^h\,\mathrm{d}x\rightharpoonup {\mathscr {U}}_{y,b,{\mathscr {M}}}\quad \text {weakly in }W^{1,2}(\omega ), \end{aligned}$$

3.11 $$\begin{aligned}&\nabla _h (u_{m^h}\circ y^h)\rightharpoonup (\nabla '{\mathscr {U}}_{y,b,{\mathscr {M}}}|{\mathscr {V}})^T\quad \text {weakly in }L^2(\varOmega ;{\mathbb {R}}^3). \end{aligned}$$

Eventually, the following liminf inequality for the energy holds true:

3.12 $$\begin{aligned} \liminf _{h\rightarrow 0} E_h(y^h,m^h)&\ge {\mathcal {F}}(y,b,{\mathscr {M}}). \end{aligned}$$

The statement of our second main result requires the specification of the class of admissible deformations. This is given through the following definition.

### Definition 3.3

(Approximately injective deformations) We define the set ${\mathcal {Y}}$ of *approximately injective deformations* as

$$\begin{aligned}&{\mathcal {Y}}:=\Big \{y\in W^{2,p}(\omega ;{\mathbb {R}}^3):\,\text {there exist }b\in W^{1,p}(\omega ;{\mathbb {R}}^3)\text { and }{\mathscr {M}}\in W^{1,2}(\omega ;\mathbb {S}^2)\text { such that }(y,b,{\mathscr {M}})\in {\mathcal {A}},\\&\quad \text {and there exists a sequence }\{f_h\}_h\subset W^{2,p}(\omega ;{\mathbb {R}}^3)\text { for which }\\&y^h(x):=y(x')+hx_3 b(x')+ f^h(x') \text { satisfy }(2.2)\text { and }h^{-2}f^h\rightarrow 0\text { strongly in }W^{2,p}(\omega ;{\mathbb {R}}^3)\text { as }h\rightarrow 0\Big \}. \end{aligned}$$

The deformations in Fig. 1 and on the right of Fig. 2 fulfill the requirements of Definition 3.3, whereas those depicted on the left of Fig. 2 and in Fig. 3 are not included in the above setting. Let us note that, although still not covering all realistic thin-film deformations, the set of approximately injective deformations encompasses a wider range of scenarios compared to those allowed by (2.10).

We provide below a construction of a recovery sequence for triples $(y,b,{\mathscr {M}})\in {\mathcal {A}}$ under the assumption that $y\in {\mathcal {Y}}$.

### Theorem 3.4

(Optimality of the lower bound for approximately injective deformations) Let $y\in {\mathcal {Y}}$ and *b* and ${\mathscr {M}}$ given by the definition of ${\mathcal {Y}}$ so that $(y,b,{\mathscr {M}})\in {\mathcal {A}}$. Then, there exists a recovery sequence $\{(y^h,m^h)\}_h\subset W^{2,p}(\varOmega ;{\mathbb {R}}^3)\times W^{1,2}(\varOmega ^{y^h};{\mathbb {R}}^3)$ such that, setting $u^h$ as the solution to the Maxwell system equation

$$\begin{aligned} \mathrm{div}\,(-\mu _0 \nabla u_{m^h}+\chi _{\varOmega ^{y^h}}m^h)=0 \end{aligned}$$

with zero mean, there holds

$$\begin{aligned}&y^h-\fint _{\varOmega }{y^h}\,\mathrm{d}x\rightarrow y\quad \text {strongly in }W^{2,p}(\varOmega ;{\mathbb {R}}^3),\\&\nabla _h y^h\rightarrow (\nabla ' y|b)\quad \text {strongly in }W^{1,p}(\varOmega ;\mathbb {M}^{3\times 3}),\\&\frac{\partial _{33}y^h}{h}\rightarrow 0\quad \text {strongly in }L^p(\varOmega ;{\mathbb {R}}^3). \end{aligned}$$

Additionally,

$$\begin{aligned}&m^h\circ y^h={\mathscr {M}}\quad \text {for every }h>0,\\&\nabla _h(m^h\circ y^h)=(\nabla '{\mathscr {M}}|0)\quad \text {for every }h>0,\\&u_{m^h}\circ y^h-\fint _{\varOmega }u_{m^h}\circ y^h\,\mathrm{d}x\rightharpoonup {\mathscr {U}}_{y,b,{\mathscr {M}}}\quad \text {weakly in }W^{1,2}(\omega ;),\\&\nabla _h (u_{m^h}\circ y^h)\rightharpoonup (\nabla '{\mathscr {U}}_{y,b,{\mathscr {M}}}|{\mathscr {V}}_{y,b,{\mathscr {M}}})^T\quad \text {weakly in }L^2(\varOmega ;{\mathbb {R}}^3), \end{aligned}$$

and the following limsup inequality for the energy holds true:

$$\begin{aligned} \limsup _{h\rightarrow 0} E_h(y^h,m^h)\le {\mathcal {F}}(y,b,{\mathscr {M}}). \end{aligned}$$

In order to give a full $\varGamma$-convergence result, in the remainder of the section we restrict our analysis to deformations satisfying the following *uniform averaged invertibility constraint*: there exists a constant $C>0$ such that

3.13 $$\begin{aligned} \Big |\int _{-\frac{1}{2}}^{\frac{1}{2}} y^h(x',x_3)\,\mathrm{d}x_3-\int _{-\frac{1}{2}}^{\frac{1}{2}} y^h(z',x_3)\,\mathrm{d}x_3\Big |\ge C|x'-z'|\quad \text {for every}\,h>0, \end{aligned}$$

for every $x',\,z'\in \omega$. Note that the condition above has a pointwise meaning because maps with uniformly bounded energies are at least $C^1$-regular.

The key idea of (3.13) is that deformed vertical fibers might intersect, but are, in average, distant enough, compared to the distance of the original points in the cross section.

Let us start by remarking that, under the same assumptions of Proposition 3.2, and assuming additionally (3.13), the limiting deformations $y\in W^{2,p}(\omega ;{\mathbb {R}}^3)$ have the additional property:

3.14 $$\begin{aligned}&\text {There exists a constant} C>0\text { such that } |y(x')-y(z')|\ge C|x'-z'|\text { for every }x',\,z'\in \omega . \end{aligned}$$

In fact, property (3.14) follows from (3.5) and (3.13). In view of (3.14), we are in the position of obtaining the following $\varGamma$-convergence result.

### Theorem 3.5

($\varGamma$-convergence under uniform averaged invertibility) Let $\{(y^h,m^h)\}\subset W^{2,p}(\varOmega ;{\mathbb {R}}^3)\times W^{1,2}(\varOmega ^{y^h};{\mathbb {R}}^3)$ be such that (2.11) and (3.13) hold true. Then, there exist $(y,b,{\mathscr {M}})\in {\mathcal {A}}$, $d\in L^p(\varOmega ;{\mathbb {R}}^3)$, and $\varepsilon >0$ satisfying (3.14), such that, up to the extraction of a (not relabeled) subsequence, there holds

$$\begin{aligned}&y^h\rightharpoonup y\quad \text {weakly in }W^{2,p}(\varOmega ;{\mathbb {R}}^3),\\&\nabla _h y^h\rightharpoonup (\nabla ' y|b)\quad \text {weakly in }W^{1,p}(\varOmega ;\mathbb {M}^{3\times 3}),\\&\frac{\partial ^2_{33}y^h}{h^2}\rightharpoonup d\quad \text {weakly in }L^p(\varOmega ;{\mathbb {R}}^3). \end{aligned}$$

Additionally, there exist $\eta \in L^2(\varOmega ;{\mathbb {R}}^3)$, and ${\mathscr {V}}\in L^2(\varOmega )$ such that $\int _{-\tfrac{1}{2}}^{\tfrac{1}{2}}{\mathscr {V}}\,\mathrm{d}x_3={\mathscr {V}}_{y,b,{\mathscr {M}}}$, and up to subsequences we have

$$\begin{aligned}&m^h\circ y^h\rightharpoonup {\mathscr {M}}\quad \text {weakly in }W^{1,2}(\varOmega ;{\mathbb {R}}^3),\\&\nabla _h(m^h\circ y^h)\rightharpoonup (\nabla '{\mathscr {M}}|\eta )\quad \text {weakly in }L^{2}(\varOmega ;\mathbb {M}^{3\times 3}),\\&u_{m^h}\circ y^h-\fint _{\varOmega }u_{m^h}\circ y^h\,\mathrm{d}x\rightharpoonup {\mathscr {U}}_{y,b,{\mathscr {M}}}\quad \text {weakly in }W^{1,2}(\omega ),\\&\nabla _h (u_{m^h}\circ y^h)\rightharpoonup (\nabla '{\mathscr {U}}_{y,b,{\mathscr {M}}}|{\mathscr {V}})^T\quad \text {weakly in }L^2(\varOmega ;{\mathbb {R}}^3). \end{aligned}$$

Eventually, the following liminf inequality for the energy holds true:

$$\begin{aligned} \liminf _{h\rightarrow 0} E_h(y^h,m^h)&\ge {\mathcal {F}}(y,b,{\mathscr {M}}). \end{aligned}$$

Conversely, for every $(y,b,{\mathscr {M}})\in {\mathcal {A}}$ with *y* satisfying (3.14) there exist $\{(\bar{y}^h,\bar{m}^h)\}_h\subset W^{2,p}(\varOmega ;{\mathbb {R}}^3)\times W^{1,2}(\varOmega ^{y^h};{\mathbb {R}}^3)$ such that, setting $u_{\bar{m}^h}$ as the solution to the Maxwell’s equation

$$\begin{aligned} \mathrm{div}\,(-\mu _0 \nabla u_{\bar{m}^h}+\chi _{\varOmega ^{\bar{y}^h}}\bar{m}^h)=0 \end{aligned}$$

with zero mean, there holds

$$\begin{aligned}&\bar{y}^h-\fint _{\varOmega }{\bar{y}^h}\,\mathrm{d}x\rightarrow y\quad \text {strongly in }W^{2,p}(\varOmega ;{\mathbb {R}}^3),\\&\nabla _h \bar{y}^h\rightarrow (\nabla ' y|b)\quad \text {strongly in }W^{1,p}(\varOmega ;\mathbb {M}^{3\times 3}),\\&\frac{\partial _{33}\bar{y}^h}{h}\rightarrow 0\quad \text {strongly in }L^p(\varOmega ;{\mathbb {R}}^3). \end{aligned}$$

Additionally,

$$\begin{aligned}&\bar{m}^h\circ \bar{y}^h={\mathscr {M}}\quad \text {for every }h>0,\\&\nabla _h(\bar{m}^h\circ \bar{y}^h)=(\nabla '{\mathscr {M}}|0)\quad \text {for every }h>0,\\&u_{\bar{m}^h}\circ \bar{y}^h-\fint _{\varOmega }u_{\bar{m}^h}\circ \bar{y}^h\,\mathrm{d}x\rightharpoonup {\mathscr {U}}_{y,b,{\mathscr {M}}}\quad \text {weakly in }W^{1,2}(\omega ;),\\&\nabla _h (u_{\bar{m}^h}\circ \bar{y}^h)\rightharpoonup (\nabla '{\mathscr {U}}_{y,b,{\mathscr {M}}}|{\mathscr {V}}_{y,b,{\mathscr {M}}})^T\quad \text {weakly in }L^2(\varOmega ;{\mathbb {R}}^3), \end{aligned}$$

and the following limsup inequality for the energy holds true:

$$\begin{aligned} \limsup _{h\rightarrow 0} E_h(\bar{y}^h,\bar{m}^h)\le {\mathcal {F}}(y,b,{\mathscr {M}}). \end{aligned}$$

A proof of the statement is in Sect. 4 below.

## Proofs

We collect in this section the proofs of the statements from Sect. 3. Within each subsection, notations are taken from the corresponding statement.

### Proof of Lemma 3.1

We first observe that by the definition of the set of admissible states ${\mathcal {A}}$ there holds

4.1 $$\begin{aligned} \mathrm{det}\,\widetilde{(\nabla 'y|b)}\ge \varepsilon \quad \text {on}\ {\mathbb {R}}^2. \end{aligned}$$

Additionally, for every $x'\in {\mathbb {R}}^2$ the matrix $(\widetilde{(\nabla 'y|b)}(x'))^{-1}(\widetilde{(\nabla 'y|b)}(x'))^{- T }$ is symmetric. By (4.1), denoting by $\lambda _i(x')$, $i=1,2,3$ the three eigenvalues of $(\widetilde{(\nabla 'y|b)}(x'))^{-1}(\widetilde{(\nabla 'y|b)}(x'))^{- T }$ in increasing order, it follows that each of them is different from zero for every $x'\in {\mathbb {R}}^2$. By the continuous dependence of the eigenvalues of a matrix on the entries of the matrix itself, and by the continuity of the map $x'\mapsto (\widetilde{(\nabla 'y|b)}(x'))^{-1}(\widetilde{(\nabla 'y|b)}(x'))^{- T }$ (see again the definition of ${\mathcal {A}}$), we deduce that for every $i=1,2,3$ there exists a point $x^i\in \bar{\omega }$ such that

$$\begin{aligned} \min _{x\in \bar{\omega }}\lambda _i(x)=\lambda _i(x^i)>0. \end{aligned}$$

Thus, recalling (3.1), we obtain

4.2 $$\begin{aligned} \min _{i=1,2,3} \min _{x\in {\mathbb {R}}^2}\lambda _i(x)=\min _{i=1,2,3} \min \{1,\lambda _i(x^i)\}{=}{:}\lambda _{\mathrm{eigen}}>0. \end{aligned}$$

As a consequence of (4.2), the quadratic form

$$\begin{aligned} Q(x,v):=(\widetilde{(\nabla 'y|b)}(x'))^{-1}(\widetilde{(\nabla 'y|b)}(x'))^{- T }v\cdot v\quad \text {for every}\,x'\in {\mathbb {R}}^2,\,v\in {\mathbb {R}}^3 \end{aligned}$$

satisfies

$$\begin{aligned} Q(x,v)\ge \lambda _{\mathrm{eigen}}|v|^2\quad \text {for every}\,x'\in {\mathbb {R}}^2,\,v\in {\mathbb {R}}^3. \end{aligned}$$

The thesis is thus a direct consequence of the uniform ellipticity of *Q*. $\square$

### Proof of Theorem 3.2

We subdivide the proof into three steps: in Step 1 we prove the compactness of sequences of deformations and magnetizations with equibounded energies. Step 2 is devoted to a characterization of the limiting stray field. Step 3 contains the proof of the liminf inequality.

**Step 1: Compactness**. In view of (2.4), (2.7), and (2.11), we infer the existence of a constant *C* such that

4.3 $$\begin{aligned}&\Vert \nabla _h y^h\Vert _{W^{1,p}(\varOmega ;\mathbb {M}^{3\times 3})}\le C,\nonumber \\&\Big \Vert \frac{1}{\mathrm{det}\,\nabla _h y^h}\Big \Vert _{L^q(\varOmega )}\le C, \end{aligned}$$

for every $h>0$. By (4.3), and by the observation that

$$\begin{aligned} \Vert \nabla y^h\Vert _{L^p(\varOmega ;\mathbb {M}^{3\times 3})}\le \Vert \nabla _hy^h\Vert _{L^p(\varOmega ;\mathbb {M}^{3\times 3})}, \end{aligned}$$

we deduce that there exists $y\in W^{2,p}(\varOmega ;{\mathbb {R}}^3)$ such that (3.5) is satisfied. In particular, by (4.3) we have $\partial _3 y=0$, thus *y* can be identified with a map in $W^{2,p}(\omega ;{\mathbb {R}}^3)$. As a further consequence of (4.3), we also find $b\in W^{1,p}(\omega ;{\mathbb {R}}^3)$ and $d\in L^p(\varOmega ;{\mathbb {R}}^3)$ such that (3.6) and (3.7) hold true. By (3.6), the continuity of $\varPhi$, and Fatou’s lemma we obtain

4.4 $$\begin{aligned} \liminf _{h\rightarrow 0}\int _{\varOmega }\varPhi (\nabla _h y^h)\,\mathrm{d}x\ge \int _{\omega }\varPhi (\nabla 'y|b)\,\mathrm{d}x',\end{aligned}$$

which implies that $\mathrm{det}\,(\nabla 'y|b)>0$ almost everywhere in $\varOmega$. Since $(\nabla 'y|b)\in W^{1,p}(\omega ;\mathbb {M}^{3\times 3})\subset C^{0,\alpha }(\bar{\omega };\mathbb {M}^{3\times 3})$ for $\alpha =(p-2)/p$, the argument in [12, Theorem 3.1] yields $(\nabla 'y|b)^{-1}\in C^0(\bar{\omega };\mathbb {M}^{3\times 3})$, $\text {det}(\nabla 'y|b)\in C^0(\bar{\varOmega })$, and $\text {det}(\nabla 'y|b)>\varepsilon$ for some $\varepsilon >0$.

From convergences (3.5)–(3.6), it follows in particular that

4.5 $$\begin{aligned} \mathrm{det}\,\nabla _h y^h\rightarrow \mathrm{det}\,(\nabla ' y|b)\quad \text {strongly in }C^0(\bar{\varOmega }), \end{aligned}$$

and hence

4.6 $$\begin{aligned} \mathrm{det}\,\nabla _h y^h\ge \frac{\varepsilon }{2}\quad \text {on }\bar{\varOmega } \end{aligned}$$

for *h* small. Properties (2.11) and (4.6) imply that

4.7 $$\begin{aligned}&\int _{\varOmega }|(\nabla m^h)\circ y^h|^2\,\mathrm{d}x\le \frac{2}{\varepsilon }\int _{\varOmega }|(\nabla m^h)\circ y^h|^2\mathrm{det}\,\nabla _h y^h\,\mathrm{d}x\nonumber \\&\quad =\frac{2}{h\varepsilon }\int _{\varOmega }|(\nabla m^h)\circ y^h|^2\mathrm{det}\,\nabla y^h\,\mathrm{d}x=\frac{2}{h\varepsilon }\int _{\varOmega ^{y^h}}|\nabla m^h|^2\,\mathrm{d}\xi \le C. \end{aligned}$$

In view of convergences (3.6) and (4.5), there holds

4.8 $$\begin{aligned} (\nabla _h y^h)^{-1}\rightarrow (\nabla 'y|b)^{-1}\quad \text {strongly in }C^0(\bar{\varOmega };\mathbb {M}^{3\times 3}), \end{aligned}$$

as well as

4.9 $$\begin{aligned} \nabla _h y^h\rightarrow (\nabla 'y|b)\quad \text {strongly in }C^0(\bar{\varOmega };\mathbb {M}^{3\times 3}). \end{aligned}$$

By combining bound (4.7) with convergence (4.9), we conclude that

4.10 $$\begin{aligned} \int _{\varOmega }|\nabla _h (m^h\circ y^h)|^2\,\mathrm{d}x\le \int _{\varOmega }|(\nabla m^h)\circ y^h|^2|\nabla _h y^h|^2\,\mathrm{d}x\le C\int _{\varOmega }|(\nabla m^h)\circ y^h|^2\,\mathrm{d}x\le C. \end{aligned}$$

In addition, by (2.11) and by the saturation constraint $|m|=1$, we deduce that

4.11 $$\begin{aligned} \int _{\varOmega }|m^h\circ y^h|^2\,\mathrm{d}x\le C. \end{aligned}$$

Estimates (4.10) and (4.11) yield the existence of maps ${\mathscr {M}}\in W^{1,2}(\omega ;\mathbb {S}^2)$ and $\eta \in L^2(\varOmega ;{\mathbb {R}}^3)$ such that convergences (3.8) and (3.9) hold, up to not relabeled subsequences. In particular, there holds

$$\begin{aligned} (\nabla m^h)\circ y^h=(\nabla _h y^h)^{-T}\nabla _h (m^h\circ y^h)\rightharpoonup (\nabla 'y|b)^{-T}(\nabla '{\mathscr {M}}|\eta )\quad \text {weakly in }L^2(\varOmega ;\mathbb {M}^{3\times 3}), \end{aligned}$$

and thus, by lower semicontinuity

4.12 $$\begin{aligned}&\alpha \int _{\varOmega }|(\nabla 'y|b)^{-T}(\nabla '{\mathscr {M}}|\eta )|^2\mathrm{det}\,(\nabla 'y|b)\,\mathrm{d}x \le \liminf _{h\rightarrow 0}\Big \{\frac{\alpha }{h}\int _{\varOmega ^y}|\nabla m|^2\Big \}. \end{aligned}$$

The boundary conditions in the definition of ${\mathcal {A}}$ are a direct consequence of (3.6). Thus, we conclude that $(y,b,{\mathscr {M}})\in ~{\mathcal {A}}$.

Regarding the compactness of the stray field, we observe that by (2.11), (4.6), and (4.9) there holds

4.13 $$\begin{aligned}&\int _{\varOmega }|\nabla _h (u_{m^h}\circ y^h)|^2\,\mathrm{d}x\le \frac{C}{h}\int _{\varOmega ^{y^h}}|\nabla u_{m^h}|^2\,\mathrm{d}\xi \le \frac{C}{h}\int _{{\mathbb {R}}^3}|\nabla u_{m^h}|^2\,\mathrm{d}\xi \le C. \end{aligned}$$

Therefore, by the Poincaré inequality we find ${\mathscr {U}}\in W^{1,2}(\omega ;{\mathbb {R}}^3)$ and ${\mathscr {V}}\in L^2(\omega ;{\mathbb {R}}^3)$ satisfying

$$\begin{aligned}&u_{m^h}\circ y^h-\fint _{\varOmega }u_{m^h}\circ y^h\,\mathrm{d}x\rightharpoonup {\mathscr {U}}\quad \text {weakly in }W^{1,2}(\omega ),\\&\nabla _h (u_{m^h}\circ y^h)\rightharpoonup (\nabla '{\mathscr {U}}|{\mathscr {V}})^T\quad \text {weakly in }L^2(\varOmega ;{\mathbb {R}}^3). \end{aligned}$$

**Step 2: the Maxwell system**. In order to show that ${\mathscr {U}}={\mathscr {U}}_{y,b,{\mathscr {M}}}$, $\int _{-\tfrac{1}{2}}^{\tfrac{1}{2}}{\mathscr {V}}\mathrm{d}x_3={\mathscr {V}}_{y,b,{\mathscr {M}}}$, and to pass to the limit in the magnetostatic energy, we observe that, since $u_{m^h}$ solves

4.14 $$\begin{aligned} \mathrm{div}\,(-\mu _0 \nabla u_{m^h}+\chi _{\varOmega ^{y^h}}m^h)=0\quad \text {in} \ {\mathbb {R}}^3, \end{aligned}$$

there holds

$$\begin{aligned}&\frac{\mu _0}{h}\int _{ {\mathbb {R}}^3 }|\nabla u_{m^h}|^2\,\mathrm{d}\xi =\frac{\mu _0}{h}\int _{\varOmega ^{y^h}}m^h\cdot \nabla u_{m^h}\,\mathrm{d}\xi \\&\quad =\frac{\mu _0}{h}\int _{\varOmega }(m^h\circ y^h)\cdot (\nabla u_{m^h})\circ y^h\, \mathrm{det}\nabla y^h\,\mathrm{d}x=\mu _0\int _{\varOmega }(m^h\circ y^h)\cdot (\nabla _h y^h)^{-T}\nabla _h (u_{m^h}\circ y^h)\,\mathrm{det}\nabla _h y^h\,\mathrm{d}x. \end{aligned}$$

Therefore, by (3.8), (4.5), (3.11), and (4.8) we conclude that

4.15 $$\begin{aligned} \lim _{h\rightarrow 0}\frac{\mu _0}{h}\int _{ {\mathbb {R}}^3}|\nabla u_{m^h}|^2\,\mathrm{d}\xi =\mu _0\int _{\varOmega }{\mathscr {M}}\cdot (\nabla 'y|b)^{-T}(\nabla '{\mathscr {U}}|{\mathscr {V}})^T\mathrm{det}(\nabla 'y|b)\,\mathrm{d}x. \end{aligned}$$

We proceed now by passing to the limit into Maxwell’s system. Denote by $\tilde{\varOmega }$ the set

$$\begin{aligned} \tilde{\varOmega }:=\mathbb {R}^2\times \big (-\tfrac{1}{2},\tfrac{1}{2}\big ), \end{aligned}$$

and consider the deformations

4.16 $$\begin{aligned} {\tilde{y}}^h(x):={\left\{ \begin{array}{ll} y^h(x)&{}\text {if}\,x\in \varOmega \\ (x',hx_3)&{}\text {if}\,x\in \tilde{\varOmega }{\setminus }\varOmega . \end{array}\right. } \end{aligned}$$

In view of (2.12), it follows that $\{{\tilde{y}}^h\}_h\subset W^{2,p}_\mathrm{loc}(\tilde{\varOmega };\mathbb {R}^3)$. Let now $\varphi \in C^{\infty }_c(\tilde{\varOmega })$. Choosing $\varphi \circ ({\tilde{y}}^h)^{-1}$ as a test function in (4.14), we obtain that

$$\begin{aligned} \frac{1}{h} \int _{\tilde{\varOmega }^{{\tilde{y}}^h}}(\mu _0\nabla u_{m^h}-m^h)\cdot \nabla (\varphi \circ ({\tilde{y}}^h)^{-1})\,\mathrm{d}\xi =0 \end{aligned}$$

for every $h>0$. By performing a change of variables, the previous equation rewrites as

4.17 $$\begin{aligned} \int _{\tilde{\varOmega }}(\nabla _h{\tilde{y}}^h)^{-1} [\mu _0(\nabla _h{\tilde{y}}^h)^{-T}\nabla _h(u_{m^h}\circ {\tilde{y}}^h)-\bar{m}^h\circ {\tilde{y}}^h]\cdot \nabla _h\varphi \, \mathrm{det}\,(\nabla _h{\tilde{y}}^h)\,\mathrm{d}x=0 \end{aligned}$$

for every $h>0$ and $\varphi \in C^{\infty }_c(\tilde{\varOmega })$, where

$$\begin{aligned} \bar{m}(\xi ):={\left\{ \begin{array}{ll} m^h(\xi )&{}\text {if}\ \xi \in \varOmega ^{y^h}\\ 0&{}\text {otherwise in}\ \tilde{\varOmega }^{{\tilde{y}}^h}. \end{array}\right. } \end{aligned}$$

By the boundary conditions in ${\mathcal {A}}$, convergences (4.8) and (4.5), and by definition (4.16), we deduce that

$$\begin{aligned}&(\nabla _h{\tilde{y}}^h)^{-1}\rightarrow \widetilde{(\nabla 'y|b)}\quad \text {strongly in}\ C^0(\tilde{\varOmega };\mathbb {M}^{3\times 3}),\\&\mathrm{det}(\nabla _h{\tilde{y}}^h)^{-1}\rightarrow \mathrm{det}\widetilde{(\nabla 'y|b)}\quad \text {strongly in}\ C^0(\tilde{\varOmega }), \end{aligned}$$

where $\widetilde{(\nabla 'y|b)}$ is the map defined in (3.1). Property (3.8) yields

$$\begin{aligned} \bar{m}^h\circ {\tilde{y}}^h\rightarrow \bar{{\mathscr {M}}}\quad \text {strongly in}\,L^2({\mathbb {R}}^2), \end{aligned}$$

with $\bar{{\mathscr {M}}}$ as in (3.2). Eventually, the same computations as in (4.13) yield

$$\begin{aligned} \int _{\tilde{\varOmega }}|\nabla _h(u_{m^h\circ {\tilde{y}}^h})|^2 \mathrm{d}x\le \frac{C}{h}\int _{{\mathbb {R}}^3}|\nabla u_{m^h}|^2 \mathrm{d}\xi \le C. \end{aligned}$$

Thus, by (3.10) and (3.11) we deduce that there exist $\tilde{{\mathscr {U}}}\in W^{1,2}({\mathbb {R}}^2)$ and $\tilde{{\mathscr {V}}}\in L^2(\tilde{\varOmega })$ such that

$$\begin{aligned}&u_{m^h}\circ {\tilde{y}}^h-\fint _{\varOmega }u_{m^h}\circ {\tilde{y}}^h\,\mathrm{d}x\rightharpoonup \tilde{{\mathscr {U}}}\quad \text {weakly in }W^{1,2}({\mathbb {R}}^2),\\&\nabla _h (u_{m^h}\circ {\tilde{y}}^h)\rightharpoonup (\nabla '\tilde{{\mathscr {U}}}|\tilde{{\mathscr {V}}})^T\quad \text {weakly in }L^2(\tilde{\varOmega };{\mathbb {R}}^3), \end{aligned}$$

with $\tilde{{\mathscr {U}}}={\mathscr {U}}$ and $\tilde{{\mathscr {V}}}={\mathscr {V}}$ almost everywhere in $\varOmega$.

Let now $\phi \in C^{\infty }_c(-\frac{1}{2},\frac{1}{2})$ and $\psi \in C^{\infty }_c({\mathbb {R}}^2)$, and for every $h>0$ consider the function $\varphi ^h(x):=\phi (hx_3)\psi (x')$ for every $x\in {\mathbb {R}}^2$. Choosing $\varphi ^h$ as a test function in (4.17) for every $h>0$, and passing to the limit as $h\rightarrow 0$, we conclude that

$$\begin{aligned}&\int _{{\mathbb {R}}^2}\widetilde{(\nabla ' y|b)}^{-1}[\mu _0 \widetilde{(\nabla ' y|b)}^{-T}\Big (\nabla ' \tilde{{\mathscr {U}}}|\int _{-\tfrac{1}{2}}^{\tfrac{1}{2}}\tilde{{\mathscr {V}}}\,\mathrm{d}x_3\Big )^T-\bar{{\mathscr {M}}}]\cdot (\nabla '\psi |0)^T\,\mathrm{det}\,\widetilde{(\nabla 'y|b)}\phi (0)\,\mathrm{d}x\\&\quad + \int _{{\mathbb {R}}^2}\widetilde{(\nabla ' y|b)}^{-1}[\mu _0 \widetilde{(\nabla ' y|b)}^{-T}\Big (\nabla ' \tilde{{\mathscr {U}}}|\int _{-\tfrac{1}{2}}^{\tfrac{1}{2}}\tilde{{\mathscr {V}}}\,\mathrm{d}x_3\Big )^T-\bar{{\mathscr {M}}}]\cdot (0 |\psi )^T\,\mathrm{det}\,\widetilde{(\nabla 'y|b)}\phi '(0)\,\mathrm{d}x=0. \end{aligned}$$

By the arbitrariness of $\phi \in C^{\infty }_c(-\frac{1}{2},\frac{1}{2})$ and $\psi \in C^{\infty }_c({\mathbb {R}}^2)$ and by a density argument, we conclude that

$$\begin{aligned} \int _{{\mathbb {R}}^2}\widetilde{(\nabla ' y|b)}^{-1}[\mu _0 \widetilde{(\nabla ' y|b)}^{-T}\Big (\nabla ' \tilde{{\mathscr {U}}}|\int _{-\tfrac{1}{2}}^{\tfrac{1}{2}}\tilde{{\mathscr {V}}}\,\mathrm{d}x_3\Big )^T-\bar{{\mathscr {M}}}]\cdot (\nabla '\psi |0)^T\,\mathrm{det}\,\widetilde{(\nabla 'y|b)}\,\mathrm{d}x=0 \end{aligned}$$

for every $\psi \in W^{1,2}({\mathbb {R}}^2)$, and

$$\begin{aligned} \int _{{\mathbb {R}}^2}\widetilde{(\nabla ' y|b)}^{-1}[\mu _0 \widetilde{(\nabla ' y|b)}^{-T}\Big (\nabla ' \tilde{{\mathscr {U}}}|\int _{-\tfrac{1}{2}}^{\tfrac{1}{2}}\tilde{{\mathscr {V}}}\,\mathrm{d}x_3\Big )^T-\bar{{\mathscr {M}}}]\cdot (0 |\psi )^T\,\mathrm{det}\,\widetilde{(\nabla 'y|b)}\,\mathrm{d}x=0 \end{aligned}$$

for every $\psi \in L^{2}({\mathbb {R}}^2)$. The identification ${\mathscr {U}}={\mathscr {U}}_{y,b,{\mathscr {M}}}$ and $\int _{-\tfrac{1}{2}}^{\tfrac{1}{2}}{\mathscr {V}}\,\mathrm{d}x_3={\mathscr {V}}_{y,b,{\mathscr {M}}}$ follows then by Lemma 3.1.

**Step 3: Liminf inequality**. By convergences (3.5)–(3.7), the liminf inequalities (4.4) and (4.12), and the continuity of *W*, we deduce that

4.18 $$\begin{aligned}&\liminf _{h\rightarrow 0} \Bigg \{\int _{\varOmega }W(\nabla _hy(x),m\circ y(x))\mathrm{d}x+\frac{\alpha }{h}\int _{\varOmega ^y}|\nabla m(\xi )|^2\mathrm{d}\xi +\int _{\varOmega }|\nabla ^2_h y(x)|^p\,\mathrm{d}x +\int _{\varOmega }\varPhi (\nabla _h y(x))\,\mathrm{d}x\Bigg \}\nonumber \\&\quad \ge \int _{\omega }W\big ((\nabla ' y|b),\,{\mathscr {M}}\big )\,\mathrm{d}x'+ \alpha \int _{\varOmega }|(\nabla 'y|b)^{-T}(\nabla '{\mathscr {M}}|\eta )|^2\mathrm{det}\,(\nabla 'y|b)\,\mathrm{d}x\nonumber \\&\qquad +\int _{\omega }\Bigg |\Bigg (\begin{array}{cc}(\nabla ')^2y&{}\nabla 'b\\ (\nabla 'b)^{ T }&{}d\end{array}\Bigg )\Bigg |^p\,\mathrm{d}x'+\int _{\omega }\varPhi (\nabla 'y|b)\,\mathrm{d}x\nonumber \\&\quad \ge \int _{\omega }W\big ((\nabla ' y|b),\,{\mathscr {M}}\big )\,\mathrm{d}x'+ \alpha \int _{\varOmega }|(\nabla 'y|b)^{-T}(\nabla '{\mathscr {M}}|0)|^2\mathrm{det}\,(\nabla 'y|b)\,\mathrm{d}x\nonumber \\&\qquad +\int _{\omega }(|(\nabla ')^2 y|^2+2|\nabla ' b|^2)^{p/2}\,\mathrm{d}x'+\int _{\omega }\varPhi (\nabla 'y|b)\,\mathrm{d}x'. \end{aligned}$$

The liminf inequality (3.12) follows by combining (4.15) with (4.18), and by recalling the characterization of the limiting stray field in Step 2. $\square$

### Proof of Theorem 3.4

The statement follows by considering the following recovery sequences

$$\begin{aligned} y^h(x',x_3):=y(x')+hx_3 b(x')+f^h(x')-\fint _{\omega }f^h(x')\,\mathrm{d}x' \end{aligned}$$

for almost every $x\in \varOmega$, and

$$\begin{aligned} m^h(\xi ):={\mathscr {M}}\circ (y^h)^{-1}(\xi ), \end{aligned}$$

for almost every $\xi \in \varOmega ^{y^h}$, where ${\mathscr {M}}$ has been identified with a function defined on the infinite cylinder of basis $\omega$ and then has been extended to the whole ${\mathbb {R}}^3$. The convergence of the energies and the identification of the limiting stray field follow arguing as in the compactness argument. $\square$

### Proof of Theorem 3.5

The compactness and liminf inequality follow by Theorem 3.2 and by checking that property (3.14) is preserved in the limit. The limsup inequality is obtained by observing that for *y* satisfying (3.14), the maps $\bar{y}^h(x):=y(x')+hx_3 b(x')$ for every $x\in {\mathbb {R}}^3$ satisfy both (3.13) and (2.2). The thesis follows by setting

$$\begin{aligned} \bar{m}^h(\xi ):={\mathscr {M}}\circ (\bar{y}^h)^{-1}(\xi ), \end{aligned}$$

for almost every $\xi \in \varOmega ^{\bar{y}^h}$, where ${\mathscr {M}}$ has been identified with a function defined on the infinite cylinder of basis $\omega$ and then has been extended to the whole ${\mathbb {R}}^3$, and by arguing as in Proposition 3.4. $\square$
